# The incidence and treatment trends of pediatric proximal humerus fractures

**DOI:** 10.1186/s12891-019-2948-7

**Published:** 2019-11-27

**Authors:** Juuli Hannonen, Hanna Hyvönen, Linda Korhonen, Willy Serlo, Juha-Jaakko Sinikumpu

**Affiliations:** Department of Children and Adolescents, Pediatric Surgery and Orthopedics, Oulu University Hospital, Medical Research Centre Oulu and PEDEGO Research Group, Oulu University, PoB 23, 90029 OYS Oulu, Finland

**Keywords:** Proximal humerus fractures, Incidence, Treatment trends

## Abstract

**Background:**

Proximal humerus fractures comprise approximately 2% of all pediatric fractures. In general, upper extremity fractures have increased in children. However, recent trends of proximal humerus fractures are not analyzed yet. The aim was to study the incidence and treatment trends of proximal humerus fractures in children.

**Methods:**

All 300 children, aged < 16 years, who suffered from a proximal humerus fracture in the catchment area of Oulu University Hospital, Finland, between 2005 and 2015, were included. Radiographs were reviewed, and patients, injuries, treatments, and outcomes were comprehensively studied. Annual incidence was based on the child population at risk, which changed between 84.500 and 88.100 in the study time.

**Results:**

The annual incidence of childhood proximal humerus fractures was mean 31.4/100,000 and no variation trend was found. The majority (92%) was treated nonoperatively, however, there was an increase of operative fixation from 0 to 16% during the study time (Difference 16, 95% CI 0.3 to 34.9%, *P* = 0.045). Bayonet displacement increased the risk of surgical fixation up to 16-fold (95% CI 4.8–51.4, *P* < 0.001) in a multivariate analysis when adjusted with other potential risk factors. Higher age was also associated with operative treatment (*P* = 0.002). The most usual recreational activities were horse riding, downhill skiing, snowboarding, and trampolining.

**Conclusion:**

Contrary to most upper extremity fractures in children, proximal humerus fractures did not increase during the long study period. However, their operative treatment increased compared to nonoperative treatment, but the evidence supporting that trend remains unclear.

## Background

Proximal humerus fractures in children comprise approximately 2% of all pediatric fractures [1]. They are usually caused by sport injuries, motor vehicle accidents, or birth trauma, while the usual mechanism of injury is hyperextension combined with external rotation of the shoulder. The fractures are either metaphyseal, which occur mostly in children 5–12 years of age [2, 3], or epiphyseal separations [4, 5]. Diagnosis is based on plain radiographs [6–11] and fractures are classified according to their severity and anatomic location [12]. Fractures involving the growth plate are classified with the Salter-Harris (SH) fracture classification [2, 13–16]. Displacement and angular deformity can be summarized using the Neer classification [3].

The proximal growth plate of the humerus is responsible for 80% of the bone’s longitudinal growth. Further, the periosteum is metabolically active in the immature skeleton [3, 12]; therefore, bone healing and spontaneous remodeling of proximal humerus fractures in children are usually good [17] and non-operative treatment preferred. However, the more displaced the fractures and the older the children are, the poorer the results will be [2, 17–19]. Persistent deformity, such as shortening, may decrease the outcome [20]. Surgical fixation has traditionally been recommended in proximal humerus fractures when closed reduction is unsatisfactory due to interposed long head of biceps tendon, deltoid muscle or capsule [21, 22], and in cases of nerve or artery injuries. Percutaneous Kirschner wire pinning is the most usual fixation in children, often combined with closed reduction. Plate and screw fixation are rarely justified; however, many surgeons recommend the elastic stable intramedullary nailing (ESIN) technique because it is stable enough and safe regarding the surrounding soft tissues [4, 23, 24]. This technique is reported to have no increased association to skin irritation or infections and the bone healing is effective and the functional outcome appears to be good [25].

Pediatric upper extremity fractures in general have increased since the beginning of the 2000s [26], but closer epidemiological description and the recent treatment trends of the proximal humerus fractures are mostly unknown. The purpose of this research was to study the local incidence and treatment trends of proximal humerus fractures in children.

## Methods

### Study design and materials

This population-based study consisted of 300 children younger than 16 years old, who had a proximal humerus fracture in the Oulu University Hospital district between 2005 and 2015. The hospital was the only pediatric trauma center in the study area and the respective children population at risk was 84.500–88.100 during the study time, according to the official statistics by *Statistics Finland*. All cases who had been diagnosed with *S42.2* in the International Classification of Diseases (ICD version 10) were included. The patients’ original hospital charts and radiographs were reviewed to confirm the diagnosis and get particulars regarding the type on fracture, treatment, and results. Type of injury, age, sex, the side of the injury, date, the day that the injury occurred, and clinical findings were studied. Patients with pathological fractures were excluded.

### Fracture and treatment type

The proximal humerus fractures were classified by the AO-classification for epiphyseal 11-E/1 or 11-E/2, metaphyseal 11-M/3, and metaphysis-diaphysis junction groups [27]. Growth plate fractures were further classified based on SH classification [2, 13–16].

Angular deformity, fracture displacement (gap), translational (ad latus) displacement, comminuted fractures, and potential shortening were analyzed in anterior-posterior, lateral, and Y-projections of the radiographs; glenohumeral joint congruency and luxation were recognized. The treatment was first classified as operative versus non-operative. Operatively treated cases were analyzed closer to determine the reduction type (closed/open) and osteosynthesis type. The type of anesthesia was recognized. Short-term outcomes and complications, as determined by the treating surgeon, were reviewed from hospital registries.

### Outcome variables

The annual incidence of proximal humerus fractures in an unselected child population and its potential changing trend were the main outcomes of the study. Secondary outcomes included the operative treatment rate and its potential change, and its associated factors, as well as injury and patients’ characteristics.

### Statistical analysis

The annual incidence was determined for 100,000 children at risk. Frequencies and proportions were reported. Year by year differences of proportions were evaluated by using the standardized normal deviate (SND) test for independent variables. Pearson’s chi-square test and Fisher’s exact test were used for categorical variables. Binary logistic and multivariate regression analysis were used to determine the risk with 95% confidence intervals (CIs) for operative proximal humeral fracture treatment according to the potential risk factors (age, gender, displacement, angular deformity, shortening, comminute fracture, and growth plate involvement). The threshold of statistical difference was set at *P* < 0.05 (5%). Data were analyzed using IBM SPSS Statistics, version 24 and StatsDirect statistical software, version 2.08.

## Results

### Annual incidence

The mean annual incidence of proximal humerus fractures in children < 16 years of age was 31.4/100,000 during the study time. There was no increasing or decreasing trend in the annual incidence from 2005 (27.2/100,000) to 2015 (28.5/100,000) (Difference 1.3, 95% CI − 17.5 to 15.2 per 100,000, *P* = 0.777). The mean incidence was 38.20 in girls and 25.35 in boys (Table 1).

**Table 1:** The annual incidence of proximal humerus fractures in children

The year	The incidence /100000
2005	27,2
2006	36,5
2007	36,5
2008	29,3
2009	30,5
2010	29,3
2011	36,9
2012	33,2
2013	30,8
2014	26,1
2015	28,5

Table shows the variation of annual incidence of proximal humerus fractures in children, aged < 16 years, in the geographic catchment area of Oulu University Hospital, Finland, during 2005–2015

### Patients’ and fracture characteristics

**Fig. 1:**
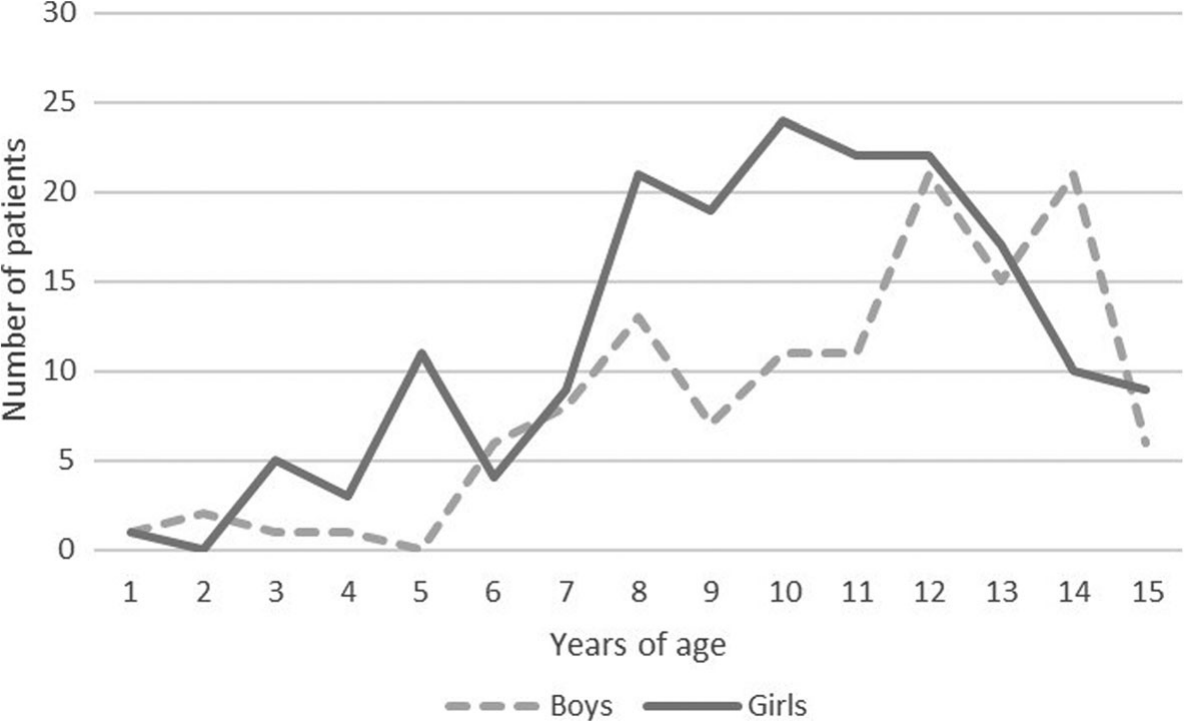
The number of fractures in boys and girls, according to age in years

There were 177 girls and 123 boys with a proximal humerus fracture. The proportion of boys was 39.1% (*N* = 9/23) in 2005 and 52% (*N* = 13/25) in 2015, respectively (Diff. 12.9, 95% CI −39.1 to 15.3%, *P* = 0.281). Their mean age was 10.2 years at the time of fracture (Fig. 1). Most of the fractures were metaphyseal (54.8%, *N* = 165), while 39.9% involved the growth plate. The following fractures were found: 11 SH type-1 fractures, 107 SH type-2, and two SH type-3 fractures. There were 15 (5.0%) proximal humerus fractures located in the meta-diaphyseal transitional zone.

### The rate of operative treatment

**Fig. 2:**
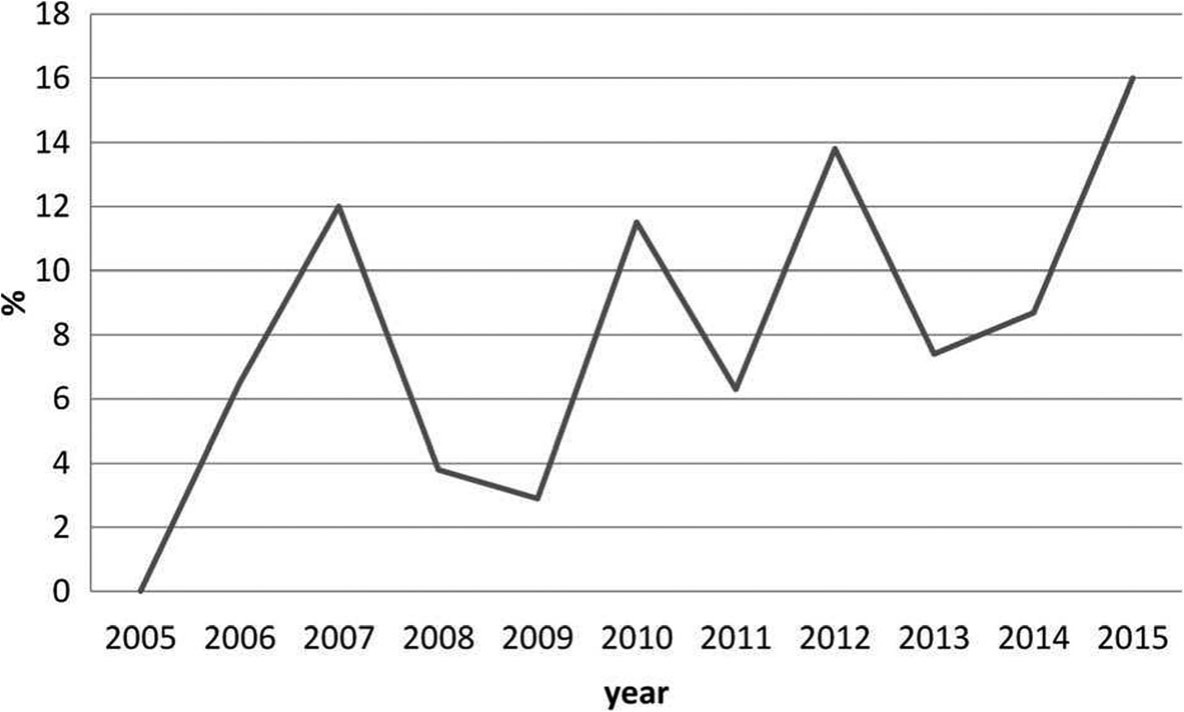
The percentage of operatively treated patients. The figure presents the percentage of the patients with proximal humerus fractures, who were treated by operative means, as compared to all cases in the same year

There was an increasing trend of operative treatment, while the rate changed from 0 to 16% during the study time (Diff. 16, 95% CI 0.3 to 34.9%, *P* = 0.045) (Fig. 2). The trend was especially seen in boys, from 5.0% in 2005–2006 to 30% in 2014–2015 (Diff. 25, 95% CI 1.6 to 48.3%, *P* = 0.049). However, a wide majority of the patients were still treated nonoperatively, three of them by closed reduction under general anesthesia without surgical fixation, while only 24 (8%) were treated operatively with surgical fixation.

More than half of the operations (58.3%, *N* = 14) were performed > 1 day after the injury, while 29.2% (*N* = 7) were operated on the next day and 12.5% (*N* = 3) were operated on the day of the injury.

### Injury types

The most usual fracture cause was riding a horse (17.3%, *N* = 52), followed by downhill skiing and snowboarding (14.0% of all, *N* = 42). Trampolining (11.0%, *N* = 33) and traffic accidents (3.3%, *N* = 10) were other common injury causes. Six injuries were ice-hockey related (2.0%). The mechanism of injury was falling on the same level in 19.3% of the cases (*N* = 58) or falling from height > 1.5 m (17.0%, *N* = 51).

### Primary complications

Ten cases, who were primarily treated nonoperatively, had to be surgically fixed later because of redisplacement. Further, one patient was re-operated after primary surgical treatment (4.2%, 1/24) due to a symptomatic scar. One in five (20.2%) of the boys suffered from complications and 14.7% of the girls (OR = 1.47, 95% CI 0.80 to 2.69; *P* = 0.216).

During the short-term follow-up of 6 months, 37 patients (12.3%) suffered from stiffness and limited range of movement, in particular, decreased shoulder rotation. Five patients suffered from persistent nerve damage, with the ulnar nerve being the most commonly injured (*N* = 8). Short-term symptoms in radial (*N* = 6), median (*N* = 5), axillary (*N* = 1), and musculocutaneous nerves (*N* = 1) were also found. Two patients (*N* = 2) showed wide plexus brachialis injuries. One of them recovered well during a further follow-up of 8 months. The rehabilitation included active physical therapy. Another plexus injury resulted in persistent morbidity.

Eight patients had a postoperative superficial fixation material infection; in seven (*N* = 7/8) of them Kirschner wires were left on the skin and in one (*N* = 1/8) the wires were left under the skin. There were in total nine patients whose Kirschner wires were left under the skin; majority of them (*N* = 8/9) recovered without any complication, while the rate of infection was 47% (*N* = 7/15) among the cases who had the wires on the skin. The percutaneous Kirschner wires which were left on top of the skin increased the risk of surgical site infection as compared with the wires left under the skin (OR = 4.20, *P* = 0.040).

### Factors affecting operative treatment

There was a gender difference in operative versus nonoperative treatment (13.7% of boys versus 4.5% of girls were operated, *P* = 0.002). Higher age was also associated with the operative treatment. One in five (20%, *N* = 13/65) children > 12 years of age were operated on compared to 1.1% (*N* = 1/87) of children < 9 years (*P* < 0.001). In the year 2005 13.0% (*N* = 3/23) of the patients were > 12 years old, and the respective number was 24.0% (*N* = 6/25) in 2015 (Diff. -11.0, 95% CI − 33.3 to 12.4%, *P* = 0.303).

The association for operative treatment increased with an increasing displacement. Patients with a fracture displacement more than a bone thickness (i.e., a bayonet displacement) were exclusively treated surgically (93.3%, *N* = 14/15); the association for surgical fixation was up to 16-fold (95% CI 4.8 to 51.4, *P* < 0.001) in the multivariate analysis when adjusted with other potential risk factors. In turn, the patients with a displacement less than half of the bone thickness were rarely surgically operated (1.3%, *N* = 2/151, *P* = 0.000). Further, angular deformity > 40° was associated with the increased association of operative treatment, but was not significant (OR = 3.12, 95% CI 0.70 to *P* = 0.13) (Table 2). Altogether 34.8% (*N* = 8/23) of the fractures were displaced > 50% of the bone thickness in year 2005. The respective rate was 48% in 2015 (*N* = 12/25) (Diff. 13.2, 95% CI − 39.1 to 14.8%, *P* = 0.274).

**Table 2:** The risk for operative treatment, according to the potential association factors

	OR	95% CI	*P*-value
Age			
< 12 years	1		
> 12 years	2.21	0.69–7.14	0.18
Gender			
Girls	1		
Boys	2.93	0.87–9.86	0.082
Angular deformity			
< 40 °	1		
> 40 °	3.12	0.70–14.51	0.13
Dislocation			
< Bone thickness	1		
> Bone thickness	15.77	4.84–51.42	< 0.001
Shortening			
< 2 cm	1		
> 2 cm	5.88	0.99–35.01	0.052
Comminuted fracture			
No	1		
Yes	3.17	0.79–12.83	0.105
Growth-plate involved			
No	1		
Yes	2.68	0.81–8.89	0.11

*OR* odds ratio, *CI* confidence interval

## Discussion

Contrary to the recent increasing trends in general pediatric upper extremity fractures [26, 28, 29], the incidence of proximal upper arm fractures has not increased during the last decade. Such different incidence trends between proximal humerus fractures and other upper extremity fractures (e.g. forearm and supracondylar humerus) is an interesting finding. The reason for the different trend of proximal humerus fractures remains unclear but can be explained by the specific injury mechanisms. Forearm and supracondylar humerus fractures were often caused by trampoline jumping [27], while proximal humerus fractures resulted from horse riding and high-energy winter sports, such as snowboarding. Greater trauma-energy may result more frequently in proximal humerus fractures than distal humerus or forearm fractures, which are usually caused by falling against the fully extended arm. From an epidemiological point of view, the number of backyard trampolines in the study area has increased since the beginning of 2000s [30, 31]; therefore, it is reasonable that trampoline related distal humerus injuries have increased [32]. However, any increase in horse riding or winter sports within the child population has not been reported in the area, to our knowledge. The annual incidence of proximal humerus fractures had no changing trend during the study period, and it was on average 31 fractures per 100,000 children every year. Proximal humerus fractures comprised approximately 2% of all fractures, compared to the total incidence of pediatric fractures (1630/100,000) in the country during the 2000s [33]. The total incidence of the proximal humerus fractures in this study was smaller than previously reported (68/100,000) by Larsen et al. in 1990 [19].

Regardless of the stable fracture incidence, surgical treatment of proximal humerus fractures had increased as an alternative to nonoperative treatment. This trend agrees the literature concerning childhood fractures in general: the operative treatment of childhood fractures has increased more than the fractures in a nationwide research of the study country [34]. Similar trend has been reported in Sweden between 1998 and 2007 [35]. However, there is no wide understanding about the recent trend of surgical treatment of proximal humerus fractures; only one recent study by Cruz et al. [36] reported an increase in the surgical treatment of proximal humerus fractures, the findings of which are strengthened by the present study. Analyzing 7520 proximal humerus fractures in the United States from 2000 to 2012, Cruz et al. found that surgical treatment increased from 39.3 to 46.4%. Nevertheless, there is sparse evidence supporting this recent change towards surgical fixation of proximal humerus fractures, and no clinical trials comparing the operative and nonoperative treatment in the modern era of fracture care are available [37].

It is generally accepted that boys suffer from bone fractures more often than girls [38]. More than 60% of all fractures affect boys [39]. Against this common trend, this study found that girls showed greater incidence of proximal humerus fractures than boys. Similar findings have been made in 2011 by Schalamon et al. [40]. Binder et al. [41] found that 50% of the patients with proximal humerus fractures were boys and girls (116). Such a gender distribution with girls being predominant, as also seen in the present study, seems extremely rare in children [42]. This is opposite to the gender distribution of corresponding fractures reported in the United States [36]. However, horse riding was found as the most common cause of injury, comprising up to 17% of all accidents in this population; since this activity is commonly considered more popular among girls, this may be one explanatory factor for the female predominance of this study. The mean age of patients suffering from proximal humerus fractures was 14 and 10 years old for boys and girls, respectively, which fits well with the general age distribution of childhood fractures [39].

For some reason, the redisplacement rate and need of later operation after primary nonoperative treatment was higher in this study than in the published literature. In 2017, Gladstein et al. [43] reported that only one patient out of 225 was re-operated on after failed nonoperative treatment. In the present study, 10 out of 286 primarily nonoperatively treated patients were surgically treated later due to a redisplacement. The difference in failed nonoperative treatment may be explained by the different clinical practice between the institutions; some surgeons may prefer nonoperative treatment primarily, and only go on to operative treatment after failed nonoperative treatment.

Primary displacement was found to be associated with operative fixation, while a bayonet position increased the risk of surgical fixation by 16-fold. Instead, even great angular deformity was not associated with increased surgical treatment. These findings are still reasonable, keeping in mind that just translational displacement (bayonet position) usually decreases the abduction motion of the shoulder, thus justifying surgical fixation [44]. In turn, angular deformity usually does not affect the functional performance of the arm, while the motion arches in shoulder joint are wide in general.

The weakness of the study was that the injury mechanism and associated background factors were not always well explained in the hospital charts. Injury history and clinical findings were based on the hospital registries. As a limitation, the number of patients who were operatively treated was not high, despite the long study period and satisfactory population at risk. A great majority of proximal humerus fractures are traditionally treated by nonoperative means. Further, there was no long-term follow-up data available, and the facture patients’ final recovery could not be determined.

The strength of this study was its inclusive population-based design: all patients in the geographic catchment area during the study period were included. There were no other pediatric trauma centers in the area and slight proximal humerus fractures were followed-up in the study center too, despite the potential first contact in primary health care. Treatment was the same for every patient despite their economic situation and possible insurance status. It is still possible that there were a few non-inhabitant patients who were treated outside the study center; however, their number must be infinitesimal.

## Conclusion

The incidence of proximal humerus fractures in children has been stable, but the rate of operative treatment, rather than nonoperative treatment, has increased during the last decade. A bayonet position associates with surgical treatment; however, the reason for increasing surgical fixation remains unclear.

## Data Availability

The data that support the findings of this study are available from the database of Oulu University Hospital, but restrictions apply to the availability of these data, which were used under license for the current study, and so are not publicly available. Data are however available from the authors upon reasonable request and with permission of Oulu University Hospital.

## Abbreviations

CI: Confidence interval
OR: Odds ratio

## References

[1] Fernandez FF, Eberhardt O, Langendorfer M, Wirth T (2008). Treatment of severely displaced proximal humeral fractures in children with retrograde elastic stable intramedullary nailing. Injury..

[2] Dameron TB, Reibel DB (1969). Fractures involving the proximal humeral epiphyseal plate. J Bone Joint Surg Am.

[3] Neer CS, Horwitz BS (1965). Fractures of the proximal humeral epiphysial plate. Clin Orthop Relat Res.

[4] Lefevre Y, Journeau P, Angelliaume A, Bouty A, Dobremez E (2014). Proximal humerus fractures in children and adolescents. Orthop Traumatol Surg Res.

[5] Kohler R, Trillaud JM (1983). Fracture and fracture separation of the proximal humerus in children: report of 136 cases. J Pediatr Orthop.

[6] van den Broek JA, Vegter J (1988). Echography in the diagnosis of epiphysiolysis of the proximal humerus in a newborn infant. Ned Tijdschr Geneeskd.

[7] Troum S, Floyd WE, Waters PM (1993). Posterior dislocation of the humeral head in infancy associated with obstetrical paralysis: a case report. J Bone Joint Surg Am.

[8] Szalay EA, Rockwood CA (1984). Injuries of the shoulder and arm. Emerg Med Clin North Am.

[9] Howard FM, Shafer SJ (1965). Injuries to the clavicle with neurovascular complications: a study of fourteen cases. J Bone Joint Surg Am.

[10] Sloth C, Just SL (1989). The apical oblique radiograph in examination of acute shoulder trauma. Eur J Radiol.

[11] Brems-Dalgaard E, Davidsen E, Sloth C (1990). Radiographic examination of the acute shoulder. Eur J Radiol.

[12] Beaty JH, Kasser JR, Beaty JH (2006). Rockwood and Wilkins’ fractures in children.

[13] Salter RB, Harris WR (1963). Injuries involving epiphyseal plates. J Bone Joint Surg Am.

[14] Peterson HA, Madhok R, Benson JT, Ilstrup DM, Melton LJ (1994). Physeal fractures: part 1. Epidemiology in Olmsted County, Minnesota, 1979–1988. J Pediatr Orthop.

[15] Fisher NA, Newman B, Lloyd J, Mimouni F (1995). Ultrasonographic evaluation of birth injury to the shoulder. J Perinatol.

[16] Burgos-Flores J, Gonzalez-Herranz P, Lopez-Mondejar JA, Ocete-Guzman JG, Amaya-Alarcon S (1993). Fractures of the proximal humeral epiphysis. Int Orthop.

[17] Shrader MW (2007). Proximal humerus and humeral shaft fractures in children. Hand Clin.

[18] Beringer DC, Weiner DS, Noble JS, Bell RH (1998). Severely displaced proximal humeral epiphyseal fractures: a follow-up study. J Pediatr Orthop.

[19] Larsen CF, Kiaer T, Lindequist S (1990). Fractures of the proximal humerus in children: nine-year follow-up of 64 unoperated on cases. Acta Orthop Scand.

[20] Pahlavan S, Baldwin KD, Pandya NK, Namdari S, Hosalkar H (2011). Proximal humerus fractures in the pediatric population: a systematic review. J Child Orthop.

[21] Dobbs MB, Luhmann SL, Gordon JE, Strecker WB, Schoenecker PL (2003). Severely displaced proximal humeral epiphyseal fractures. J Pediatr Orthop.

[22] Bahrs C, Zipplies S, Ochs BG (2009). Proximal humeral fractures in children and adolescents. J Pediatr Orthop.

[23] Sessa S, Lascombes P, Prevot J, Gagneux E, Blanquart D (1990). Centro-medullary nailing in fractures of the upper end of the humerus in children and adolescents. Chir Pediatr.

[24] Shore BJ, Hedequist DJ, Miller PE, Waters PM, Bae DS (2015). Surgical management for displaced pediatric proximal humeral fractures: a cost analysis. J Child Orthop.

[25] Canavese F, Athlani L, Marengo L (2014). Evaluation of upper-extremity function following surgical treatment of displaced proximal humerus fractures in children. J Pediatr Orthop B.

[26] Sinikumpu JJ, Lautamo A, Pokka T, Serlo W (2012). The increasing incidence of pediatric diaphyseal both-bone forearm fractures and their internal fixation during the last decade. Injury..

[27] Slongo TF, Audige L, AO Pediatric Classification Group (2007). Fracture and dislocation classification compendium for children: the AO padiatric comprehensive classification of long bone fractures (PCCF). J Orthop Trauma.

[28] Sinikumpu JJ, Victorzon S, Antila E, Pokka T, Serlo W (2014). Nonoperatively treated forearm shaft fractures in children show good long-term recovery. Acta Orthop.

[29] Ryan LM, Teach SJ, Searcy K (2010). Epidemiology of pediatric forearm fractures in Washington, DC. J Trauma.

[30] Sinikumpu JJ, Salokorpi N, Suo-Palosaari M, Pesälä J, Serlo W (2016). Severe trampoline injuries and their risk factors among children and the young. Duodecim.

[31] Königshausen M, Gothner M, Kruppa C (2014). Trampoline-related injuries in children: an increasing problem. Sportverletz Sportschaden.

[32] Korhonen L, Salokorpi N, Suo-Palosaari M, Pesälä J, Serlo W, Sinikumpu JJ (2018). Severe trampoline injuries: incidence and risk factors in children and adolescents. Eur J Pediatr Surg.

[33] Mäyränpää MK, Mäkitie O, Kallio PE (2010). Decreasing incidence and changing pattern of childhood fractures: a population-based study. J Bone Miner Res.

[34] Helenius I, Lamberg TS, Kääriäinen S, Impinen A, Pakarinen MP (2009). Operative treatment of fractures in children is increasing: a population-based study from Finland. J Bone Joint Surg Am.

[35] Hedström EM, Svensson O, Bergström U, Michno P (2010). Epidemiology of fractures in children and adolescents. Acta Orthop.

[36] Cruz AI, Kleiner JE, Gil JA, Goodman AD, Daniels AH, Eberson CP (2018). Inpatient surgical treatment of pediatric proximal humerus fractures between 2000 and 2012. J Child Orthop.

[37] Chaus G, Carry P, Pishkenari A, Hadley-Miller N (2015). Operative versus nonoperative treatment of displaced proximal humeral physeal fractures: a matched cohort. J Pediatr Orthop.

[38] Lyons RA, Delahunty AM, Kraus D (1999). Children’s fractures: a population based study. Inj Prev.

[39] Mäyränpää MK (2012). Fractures in children: epidemiology and associated bone health characteristics.

[40] Schalamon J, Dampf S, Singer G (2011). Evaluation of fractures in children and adolescents in a level I trauma center in Austria. J Trauma.

[41] Binder H, Tiefenboeck T, Payr S, Schurz M, Aldrian S, Sarahrudi K (2016). Treatment of proximal humerus fractures in children and young adolescents. Wien Klin Wochenschr.

[42] Koga H, Omori G, Koga Y, Tanifuji O, Mochizuki T, Endo N (2018). Increasing incidence of fracture and its sex difference in school children: 20 year longitudinal study based on school health statistic in Japan. J Orthop Sci.

[43] Gladstein AZ, Schade AT, Howard AW, Camp MW (2017). Reducing resource utilization during non-operative treatment of pediatric proximal humerus fractures. OTSR..

[44] Schwendenwein E, Hajdu S, Gaebler C, Stengg K, Vécsei V (2004). Displaced fractures of the proximal humerus in children require open/closed reduction and internal fixation. Eur J Pediatr Surg.

